# Dendrimer Prodrugs

**DOI:** 10.3390/molecules21060686

**Published:** 2016-05-31

**Authors:** Soraya da Silva Santos, Elizabeth Igne Ferreira, Jeanine Giarolla

**Affiliations:** Laboratory of Design and Synthesis of Chemotherapeutics Potentially Active in Neglected Diseases (LAPEN), Department of Pharmacy, Faculty of Pharmaceutical Sciences, University of São Paulo—USP, Avenue Professor Lineu Prestes, 580–Building 13, São Paulo SP, 05508-900, Brazil; soraya.ssantos@yahoo.com.br (S.S.S.); elizabeth.igne@gmail.com (E.I.F.)

**Keywords:** dendrimer, prodrugs, molecular modifications, targeted dendrimer, pharmaceutical applications

## Abstract

The main objective of this review is to describe the importance of dendrimer prodrugs in the design of new drugs, presenting numerous applications of these nanocomposites in the pharmaceutical field. Therefore, the use of dendrimer prodrugs as carrier for drug delivery, to improve pharmacokinetic properties of prototype, to promote drug sustained-release, to increase selectivity and, consequently, to decrease toxicity, are just some examples of topics that have been extensively reported in the literature, especially in the last decade. The examples discussed here give a panel of the growing interest dendrimer prodrugs have been evoking in the scientific community.

## 1. Introduction

The term “prodrug” refers to a bioreversible compound, which, *in vivo*, by chemical or enzymatic action, releases an active substance in the biological environment. This kind of interesting strategy can improve biopharmaceutical, pharmacokinetic and, indirectly, pharmacodynamics features of a prototype [1,2,3,4,5]. On the other hand, dendrimers represent an emerging class of well-defined branched macromolecules, which possess three main components: (1) a multifunctional core, which allows the attachment of branches, (2) repeated branches from the central core and (3) peripheral functionalities [6,7,8]. Dendrimer synthesis, which can be performed in a convergent, divergent or orthogonal way, is carried out step by step and the linkage of a new branch leads to a different generation [9].

As drug carriers, dendrimers can promote controlled and/or targeted drug delivery [10,11]. The bioactive compound may be conjugated to the dendrimer via [12,13]: (1) directed conjugation to the surface; (2) the interaction through a spacer group; (3) branches with drug/bioactive compound, providing an exponential increase of the active part in each subsequent generation [14,15]. Many dendrimer prodrugs have been synthesized, focusing of different biological applications, such as anticancer, anti-inflammatory and antimicrobial activity. Bioactive molecules have been conjugated to poly(amidoamine) (PAMAM), poly(propylene imine) (DAB or PPI) and poly(etherhydroxylamine) (PEHAM) dendrimers [13].

## 2. General Applications of Dendrimer Prodrugs

### 2.1. Drug Delivery

A typical dendrimer application in the drug delivery is its use as drug carrier. In this context, Jevprasesphant and colleagues [16] published an interesting review reporting the use of dendrimers as oral drug carriers, since those compounds can cross intestinal epithelial cells [17,18,19]. Considering that the mechanism of G3 PAMAM dendrimer transport has been investigated, the results concerning Caco-2 cell assays revealed the uptake of PAMAM dendrimer by endocytosis-mediated cellular internalization. Furthermore, other studies showed that surface changes (such as inclusion of lauroyl moieties or PEG chains) decrease the cytotoxic effect of these derivatives [20,21,22,23,24].

Interesting dendrimer prodrugs were designed with the goal of crossing the blood brain barrier. The first one, reported by Bhadra and coworkers [25], comprised PEGylated G4 PAMAM dendrimer conjugated to 5-fluorouracil. Another study with PEGylated G4 PAMAM dendrimers showed low *in vivo* toxicity due to high brain permeation and reduction of biodistribution in peripheral organs [26].

**Figure 1:**
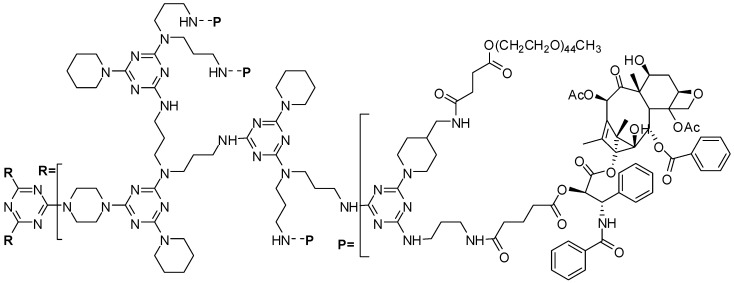
G3 Triazine dendrimer prodrug containing PEG and paclitaxel [27].

A triazine dendrimer G3 generation was conjugated to paclitaxel [27] (Figure 1). The physicochemical properties, the *in vitro* and *in vivo* efficacy and toxicity were evaluated showing good results. This is a further successful example of dendrimer application as a drug delivery system (Figure 1) [27].

Lee and coworkers [28] published a patent regarding multifunctional dendrimer prodrugs, constructed with PAMAM dendrimers and cyclodextrin. The carriers were able to transport one or more therapeutic molecules. Concerning the synthesis and drug delivery features, the authors reported several advantages, such as: (1) low cytotoxicity and high efficiency of gene transfection; (2) higher yields and purity; (3) improvement of water solubility and (4) the ability to deliver different molecules, thus achieving synergistic effects.

**Figure 2:**
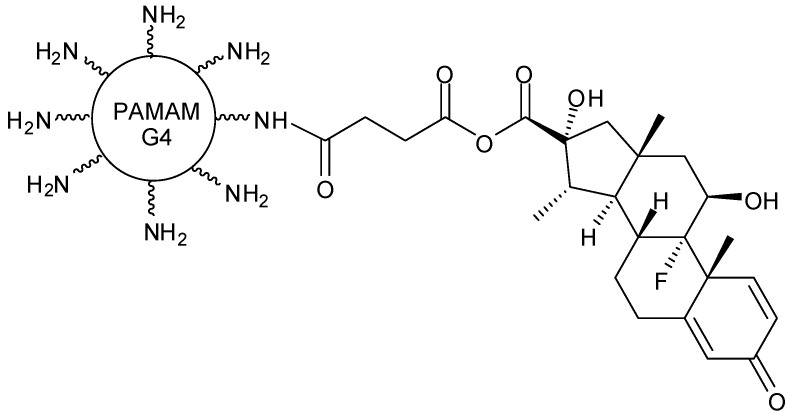
G4 PAMAM dendrimer conjugate with dexamethasone [29].

Two different dendrimers have been developed as nanocarriers for transporting dexamethasone. Those prodrugs were compared to encapsulation-based liposome delivery systems (Figure 2). The findings showed G4 PAMAM dendrimer as the most promising carrier, when conjugated to drugs, since these molecules presented higher tumor necrosis factor (TNF-alpha) inhibition [29].

**Figure 3:**
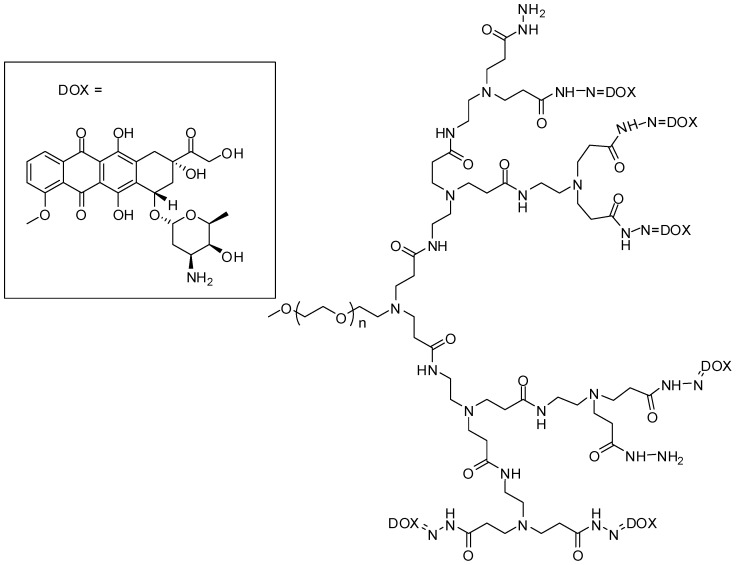
Doxorubicin PEGylated dendrimer prodrug [30].

PEGylated amphiphilic linear-dendritic prodrugs (MPEG-b-PAMAM-DOX) conjugated with doxorubicin (DOX) were synthesized by acid-labile hydrazone bonds (Figure 3). Amphiphilic compounds allow the encapsulation of hydrophobic substances as a co-delivery strategy for cancer treatment. In this context, in the hydrophobic cavity of MPEG-b-PAMAM-DOX 10-hydroxy-camptotecin was encapsulated. The release and cellular reuptake assays of both drugs was shown to be pH dependent (increased release with decrease of pH). Additionally, the molecules were effectively taken up by MCF-7 cells (a breast cancer cell model) and MPEG-b-PAMAM-DOX proved to be able to suppress cell growth more efficiently than DOX prodrug, MPEG-b-PAMAM-DOX physical mixture, camptothecin or parent drugs. Therefore, camptotecin encapsulation in DOX conjugated prodrugs can be a promising approach for drug delivery in anti-cancer treatment [30].

Dual stimulus-responsive polyplex micelles were designed by the complexation between two different types of copolymer blocks: (1) mildly acidic pH-responsive charge conversional function and (2) self-assembled anionic blocks, such as poly(ethylene glycol)-poly[(*N*′-dimethylmaleoyl-2-aminoethyl)aspartamide] (PEG-PAsp(EDA-DM)), and platinum(IV)-conjugated cationic poly(amidoamine) (PAMAM–Pt(IV)) dendrimer prodrugs. PAMAM–Pt(IV) prodrugs presented deep uptake and good dispersion activity in the tumor tissue, due to their small size and high mobility. This deep tumor tissue penetration makes this kind of drug delivery platform also interesting to overcome drug resistance [31,32].

### 2.2. Improvement of Pharmacokinetics

Dendrimer prodrugs are extremely useful for improving drug pharmacokinetics. D’Emanuele and coworkers [33] published an interesting paper based on this approach. The researchers synthesized a dendrimer derivative to increase the oral bioavailability of poorly soluble drug propranolol, known as substrate of the P-glycoprotein (P-gp) efflux transporter. The molecules were designed with PAMAM generation 3 or lauroyl-G3 PAMAM, containing two, four or six drugs. The results showed the success of this methodology, since a significant improvement of apical to basolateral transport (A→B P_app_) and reduced basolateral to apical transport (B→A P_app_) across Caco-2 cell monolayers was observed. Najlah and colleagues [34] explored the same approach, using PAMAM G1 dendrimer as drug carrier to improve the pharmacokinetic profile of terfenadine. The prodrugs were designed using G1 PAMAM, succinic acid or succinyl-diethylene glycol as a spacer and the drug. Dendrimer derivatives were more hydrophilic than the prototype. In addition, the authors pointed out the importance of prodrug design for the enhancement of oral bioavailability, since the improvement of permeability in Caco-2 monolayers assays was observed.

**Figure 4:**
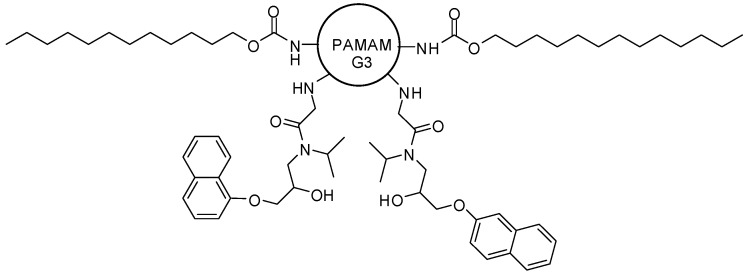
G3 PAMAM dendrimer prodrugs containing propranolol with surface changes [35].

The importance of the lauroyl moiety was also investigated by Saovapakhiran and colleagues [35]. The mechanism of internalization of G3 PAMAM dendrimer (G3), G3 PAMAM containing two lauroyl chains (G3L2), G3 PAMAM with two propranolol molecules (G3P2), G3 PAMAM composed of two lauroyl chains and two propranolol molecules (G3L2P2) was studied in the human colon adenocarcinoma HT 29 cell line (which can internalize compounds via clathrin-mediated endocytosis, caveolae-mediated endocytosis and macropinocytosis) (Figure 4). The type of surface groups on dendrimer internalization can define chemical changes to increase cellular uptake. The entrance of G3 and G3P2 involves caveolae-dependent endocytosis and macropinocytosis pathways. G3L2P2 occurs by caveolae-dependent, and possibly clathrin-dependent endocytosis pathways. G3L2 internalization comprises via caveolae-dependent, clathrin-dependent and macropinocytosis. Therefore, the lauroyl moiety increased the intracellular uptake rate, as well as it enhanced the kinetics of internalization in human intestinal epithelial cells.

**Figure 5:**
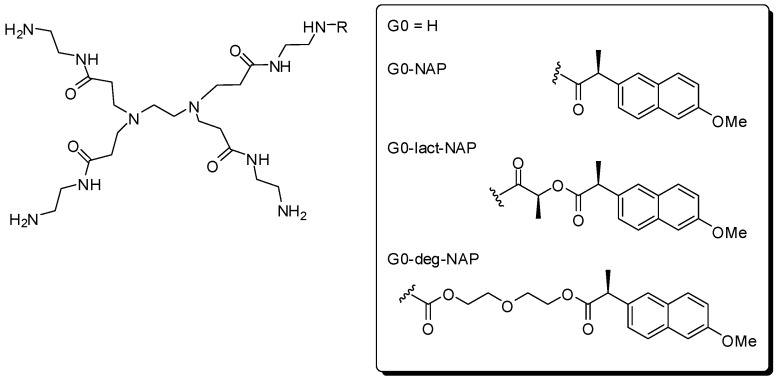
Dendrimer prodrugs useful for improving naproxen solubility and bioavailability [36].

PAMAM generation 0 was used as carrier to improve solubility, as well as bioavailability of naproxen [36]. The drug was attached in the dendrimer directly or via a spacer group (Figure 5). The success of this prodrug design was confirmed by the increase of solubility, analysed by drug partitioning between 1-octanol and phosphate buffer (pH 7.4). All derivatives showed to be stable in chemical stability assays. Additionally, the drug was released from dendrimer in the enzymatic experiments, in which spacer groups with longer chain presented faster cleavage profile.

**Figure 6:**
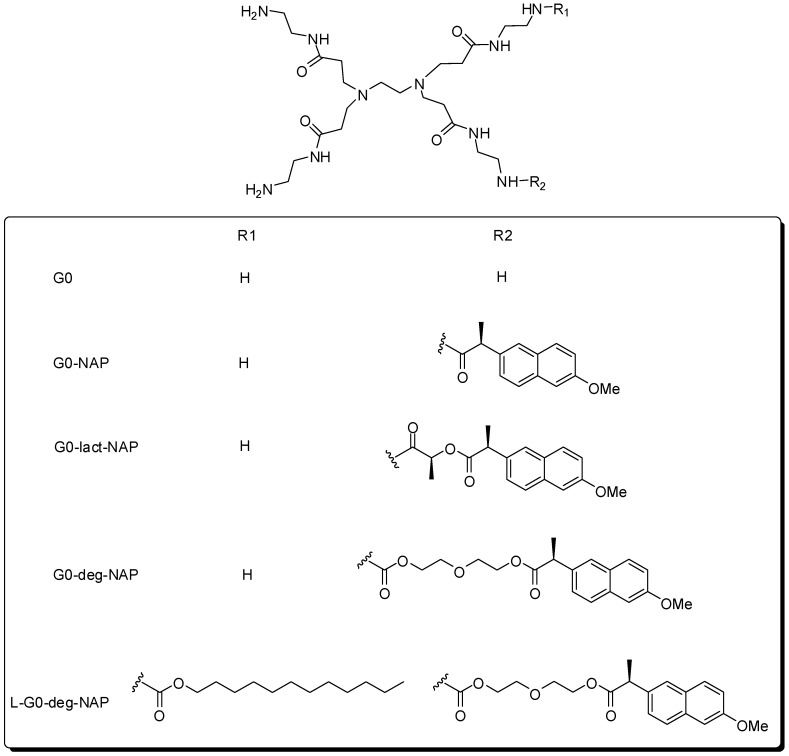
Dendrimer prodrugs containing naproxen [37].

Najlah and coworkers [37] continued the investigation of the pharmacokinetic properties of naproxen with compounds linked through ester or amide linkages to G0 PAMAM (see Figure 6). The best permeability result was observed for the compound containing ester bond and one lauroyl chain attached to the dendrimer surface (L-G0-deg-NAP). In addition, the amide derivative showed the highest stability in plasma and liver homogenate. The authors observed that the length of the chain spacer group was crucial for the prodrug hydrolysis profile. The lactate derivatives were more stable under all conditions tested. On the other hand, compounds containing diethylene glycol exhibited chemical stability, despite their rapid cleavage in plasma and liver homogenate.

The pharmacokinetic profile of doxorubicin was improved using a dendrimer prodrug approach [13,38]. According to the authors, the biodegradable polyester dendrimer conjugate showed “better blood circulation time through size and molecular architecture, drug loading through multiple attachment sites, solubility through poly(ethylene glycol) (PEG) chains, and drug release through the use of pH-sensitive hydrazone linkages”. Additionally, it is important to emphasize that the hydrolysable compound showed 10 times less toxicity in C-26 colon carcinoma cells, when compared with the parent drug. PEG has been associated with dendrimers, typically conjugated at the surface, to improve their water solubility, biocompatibility and biodistribution [39,40,41,42].

### 2.3. Drug Release Control

**Figure 7:**
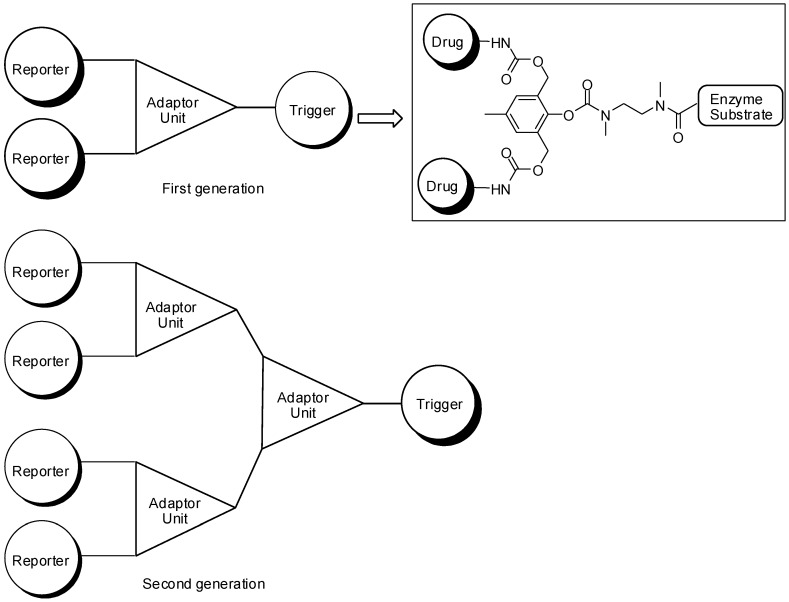
Self-immolative dendrimers [43,44].

The Shabat research group introduced the interesting concept of “self-immolative dendrimers” as novel drug delivery platforms [43]. These compounds were designed with a trigger, which can start a sequence of dendrimer fragmentations in a self-immolative manner, leading to spontaneous release of the tail units (see Figure 7). Additionally, the approach can be used as a platform to design multi-prodrugs, which may release dendrons through self-immolative chain fragmentation by single enzymatic action. Based on the foregoing considerations, different types of dendrimer prodrugs have been designed and synthesized, for instance, those with doxorubicin (DOX) and camptothecin as tail unit, and a retro-aldol retro-Michael focal point, as trigger. Catalytic antibody ^38^C2 action could start the dendrimer disassembly [44,45] (see Figure 7). The biological assays showed improvement of prodrug activity in a Molt-3 leukemia cell line. Other different and remarkable “self-immolative dendrimers” approaches were explored by the group [46,47,48,49,50,51].

**Figure 8:**
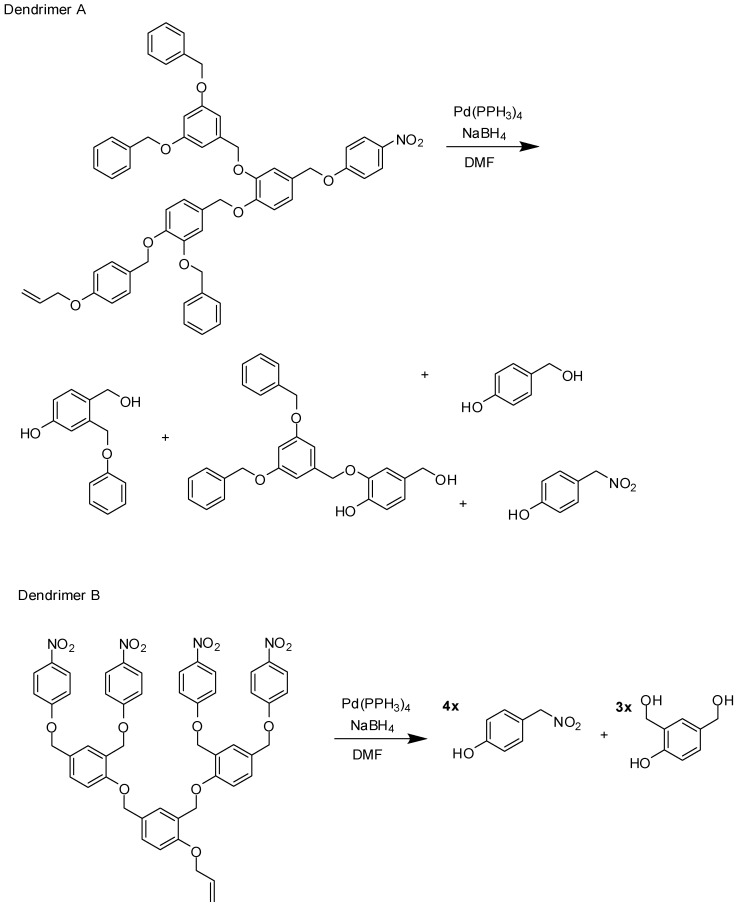
Chemical disassembly of second generation dendrimers [52].

The same dendrimer disassembly approach was explored by McGrath and coworkers [52]. The authors designed second dendrimer generations composed by hydroxybenzyl alcohol derivatives. After the cleavage of a peripheral 4-allyloxybenzyl ether group, three reactions occur, culminating in 4-nitrophenol release (Figure 8).

Salicylic acid was used to build dendritic prodrugs, aiming at extending drug release with consequent improvement of compound effectiveness [15]. Generations one to three were synthesized and the chemical structures were confirmed by Fourier Transform Infrared Spectroscopy (FTIR), ^1^H- and ^13^C-NMR, and Matrix Assisted Laser Desorption Ionization, Time of Flight, Mass Spectrometry (MALDI-TOF-MS). The prodrugs presented high purity and monodispersity, confirmed by gel-permeation chromatography.

**Figure 9:**
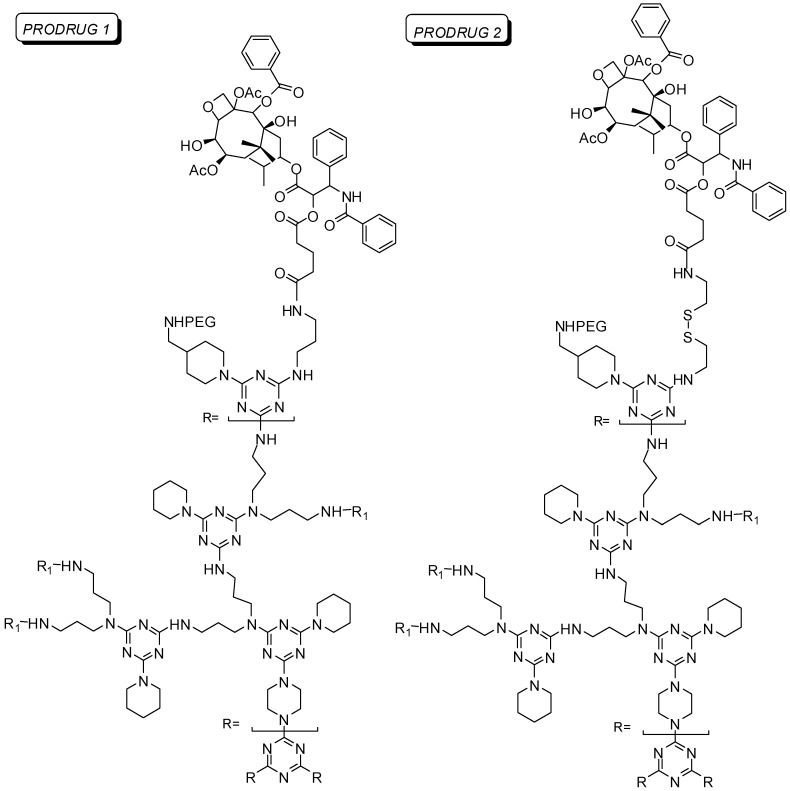
Triazine dendrimer prodrug containing paclitaxel and PEG chain [54].

Lim and colleagues [53,54] have synthesized paclitaxel dendrimer prodrugs with antitumor activity in human prostate cancer. The main objective was to analyze the influence of different biodegradable chemical bonds on the rate of drug release. Prodrugs were built up with triazine dendrimers containing ester, amide and disulfide labile bonds. PEGylation was carried out to improve water solubility, biocompability and plasma half-life. HexaPEGylated, nonaPEGylated and dodecaPEGylated compounds were synthesized (Figure 9). The drug release at cellular level was improved in prodrug 2 (disulfide bond) due to the cleavage by endogenous reductant agents, such as glutathione. In addition, this molecule was more effective than the corresponding prodrug 1.

Erythromycin is a well-known antibiotic, but its anti-inflammatory action is also attracting interest from the scientific community [55]. Considering that, and with the objective of obtaining extended drug release, among other effects, dendrimer prodrugs containing ester bonds between the drug and G4-OH PAMAM, were synthesized and characterized. Around 90% of erythromycin was released over a period of 10 h. In addition, the polymeric compound was not cytotoxic to macrophages, showed better efficacy when compared with the free molecule and presented significant reductions of nitrite levels. The antibacterial activity was similar to that of the parent drug. Therefore, a good prototype for sustained and targeted delivery was obtained.

**Figure 10:**
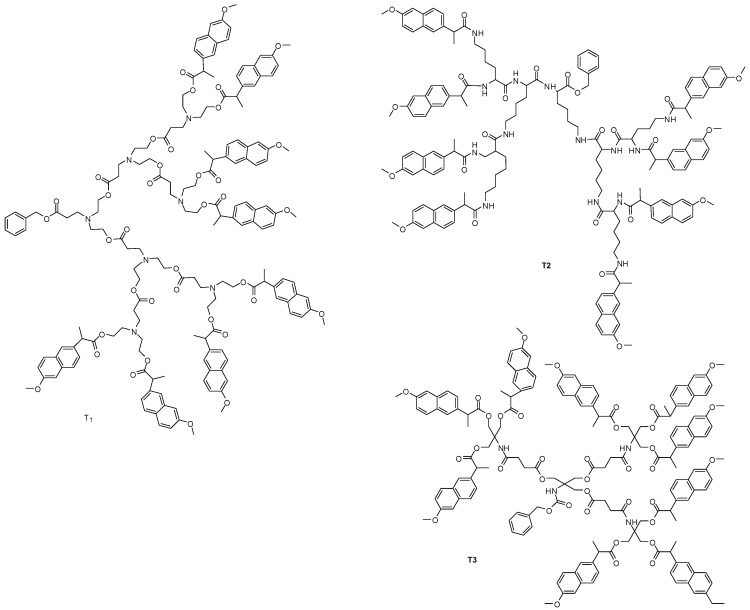
Potentially anti-inflammatory dendrimer prodrugs [56].

Three different dendrimer derivatives containing naproxen have been synthesized as potential nonsteroidal anti-inflammatory prodrugs (Figure 10). These self-immolative NSAIDs dendrimer prodrugs were planned to release multiple molecules of the naproxen after single step of enzymatic activation. Furthermore, the molecules exhibited significant anti-inflammatory activity, no relevant cytotoxicity against HEK293 cells and, lower degree of *in vivo* ulcerogenic toxicity, when compared with the parent drug [56].

Eight ciprofloxacin molecules were attached in an interesting dendrimer designed with piperazine and 1,3,5-triazine [57]. The compound was characterized by MALDI-TOF MS, NMR and IR. Additionally, the prodrug was assessed against different bacterial species, for instance, *Staphylococcus aureus*, *Bacillus subtilis*, *Escherichia coli*, *Pseudomonas aeruginosa* and *Proteus mirabilis*. The compounds also showed a pH-dependent drug release profile.

### 2.4. Molecular Modeling as a Tool to Predict the Drug/Bioactive Compound Release

Molecular modeling was applied by our group as a promising computational tool in understanding the disassembly of potentially anti-Chagas and leishmanicidal dendrimer prodrugs. 

**Figure 11:**
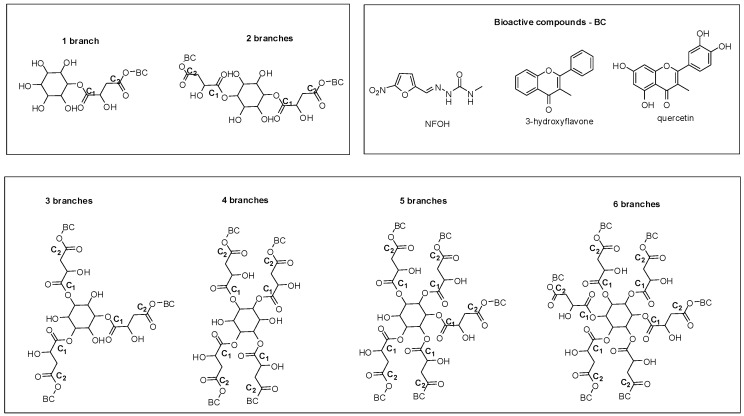
Dendrimer prodrugs for neglected diseases containing 3-hydroxyflavone, NFOH and quercetin [58].

Dendrimers were designed with *myo*-inositol (core), l-malic acid (spacer), and three different bioactive compounds: (1) hydroxymethylnitrofurazone (NFOH); (2) quercetin or (3) 3-hydroxy-flavone (Figure 11). 

Properties related to dendrimer disassembly, such as: (a) spatial carbonyl availability; (b) electronic distribution observed in the map of electrostatic potential (MEP) and (c) the lowest unoccupied molecular orbital energy (ELUMO) were evaluated. The region most susceptible to undergo a chemical or enzymatic action was the carbonyl next to the active compound for dendrimers containing one, two and three branches. Dendrimeric prodrugs with four, five and six branches revealed that carbonyl group near the core were the most promising ester breaking point [58,59]. The same approach was analyzed by Santos and colleagues [60,61]. Therein, the authors evaluated the disassembly of targeted dendrimer useful against leishmaniasis.

Another molecular modeling study concerning dendrimer prodrugs was performed by Ferreira’s group [62]. Thermodynamics, steric, steric/electronic, electronic and hydrophobic properties were calculated and Hierarchical Cluster Analysis (HCA—discriminates samples through similarity indices) and Principal Component Analysis (PCA—uses linear combinations from the original data) were carried out with the main objective of understanding the molecular systems. The results showed steric, intrinsic/steric, steric/electronic, steric/hydrophobic, hydrophobic, and electronic properties as important features to those compounds. Furthermore, these findings may contribute to elucidation of chemical and enzymatic hydrolysis mechanism, useful for understanding the bioactive compound release from dendrimer matrix. It is worth noting that despite the usefulness of this methodology to predict the drug delivery from dendrimer prodrugs, those were the only papers found in the literature.

### 2.5. Toxicity Decrease and Drug Selectivity Increase

**Figure 12:**
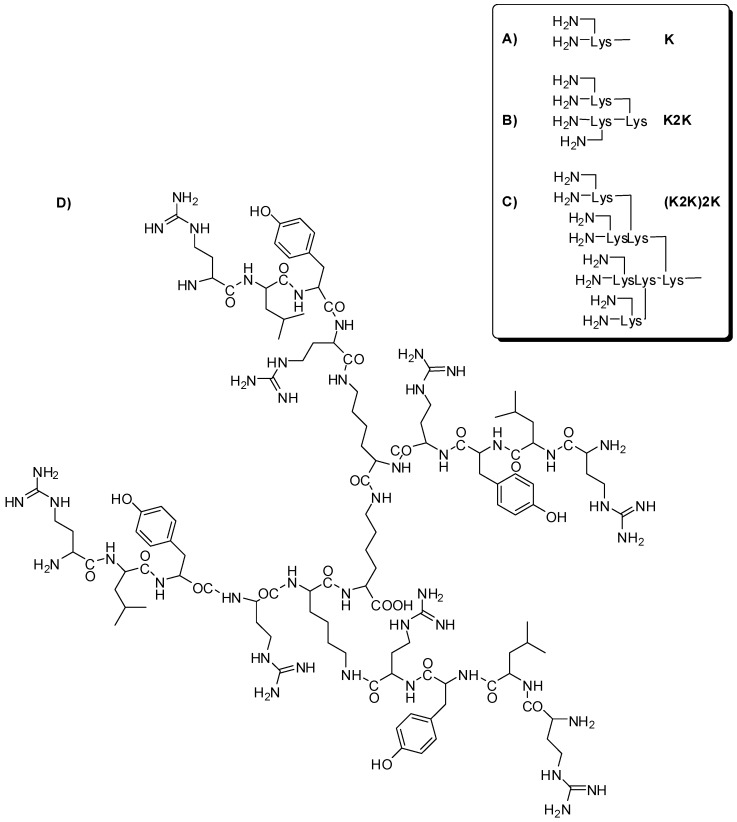
Peptide dendrimers for selective action in microbial surface [63].

Selective peptide dendrimers were designed for microbial surface action. The compounds were composed of lysine (asymmetrical core), branches containing tetrapeptides (with recognition features for microbial surface) and octapeptides (tetrapeptide duplication) (Figure 12). Peptide dendrimer activity was evaluated on ten different kinds of microorganisms and it was shown to be as active as linear peptides. Besides that, these compounds demonstrated lower toxicity and higher proteolytic resistance than the corresponding linear peptide. Moreover, small dendrimers are revealed to have lower immunogenic action than larger ones [63].

**Figure 13:**
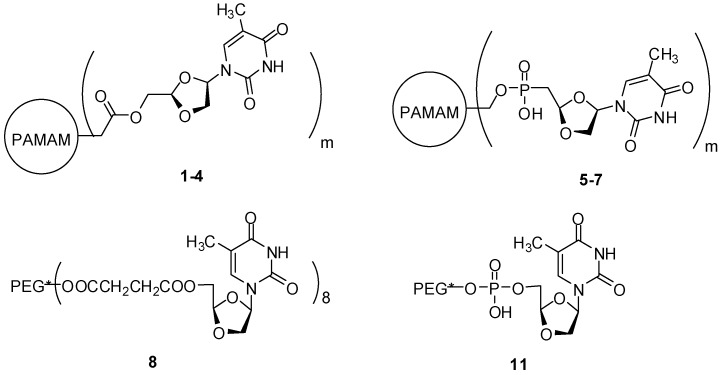
Dendrimer prodrugs potentially active in HIV [64].

Liang and coworkers [64] evaluated the anti-HIV activity of PAMAM derivatives (the authors designed the compounds using (−)-β-d-(2*R*,4*R*)-dioxolane-thymine (a bioactive molecule, named as DOT), PAMAM generations 2.0, 3.0, 5.0 and 6.0, either with or without polyethylene glycol (PEG). The prodrugs were obtained by ester or phosphate linkages, purified by applying exclusion chromatography and characterized using NMR and MALDI-TOF mass spectrometry. Almost a 140 fold-increase of anti-HIV action was obtained, resulted in the series of dendrimers synthesized via ester bond, mainly with phosphate linker (compounds **1**–**7**, Figure 13). However, these prodrugs exhibited an improvement of the toxic effects in the cytotoxicity assay. Additionally, PEG-DOT derivatives (**8**, **11**), did not present significant antiviral activity (Figure 13). Compound **1** was the best of the series and compound **5** was also effective. Based on the results, the authors could conclude that the most promising chemical architecture is PAMAM generation 2 and phosphate groups.

**Figure 14:**
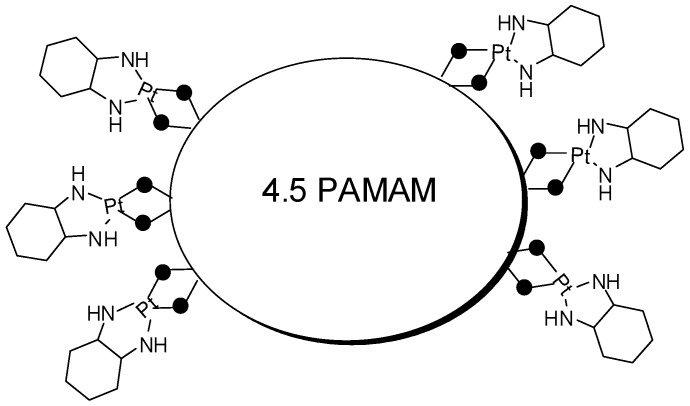
Polymeric prodrug potentially active in cancer [65].

The achievement of less toxic and more selective compounds is the major challenge in cancer drug discovery. Organoplatinum drugs, such as cisplatin and carboplatin, are effective against different types of tumors, however these molecules are toxic. Therefore, in an attempt to decrease the side effects, prodrugs with 4.5 PAMAM and diaquo(1,2-diaminocyclohexane)platinum(II) were prepared (Figure 14) [65]. The resulting synthetic compound presented 40 drugs attached to the dendrimer periphery, showing extended release to 24 h, contributing, probably, to enhance the drug effectiveness.

**Figure 15:**
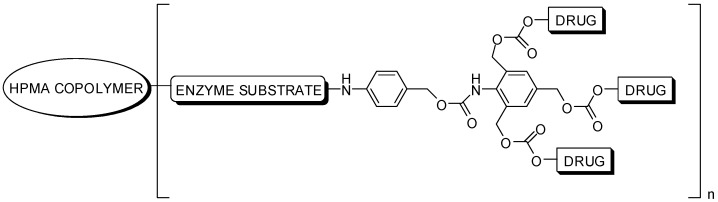
Polymer-drug conjugate designed to cancer therapy [66].

*N*-(2-Hydroxypropyl)-methacrylamide (HPMA) copolymer-drug conjugate with an AB_3_ self-immolative dendritic linker was synthesized and the biological activity was evaluated [66]. The targeted drug designed considered the selective paclitaxel released by the enzyme cathepsin B. The corresponding water-soluble molecule is presented in Figure 15, and the interesting results showed an improvement of cytotoxicity on murine prostate adenocarcinoma (TRAMP C2) cells, compared to a classic monomeric drug-polymer conjugate.

**Figure 16:**
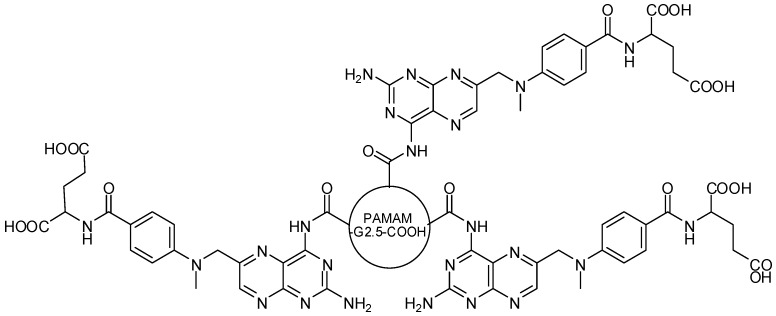
Dendrimer prodrug designed with PAMAM-G2.5-COOH-methotrexate [67].

PAMAM-methotrexate prodrugs were synthesized by Gurdag and coworkers [67], using PAMAM-G2.5-COOH and PAMAM-G3-NH_2_ dendrimers, focused on -NH_2_ or -COOH drug moiety (Figure 16). The main objective of this prodrug design was to evaluate the significance of the functional groups involved in the conjugation and the surface charge in the antitumor activity. The compounds showed significant activity and no cytotoxicity in methotrexate-resistant cancer cells [13,67]. Duncan and colleagues also synthesized antitumor dendrimer prodrugs, such as molecules containing cisplatin conjugate to G3.5 PAMAM carboxyl-terminated dendrimer. Prodrug revealed improvement in water solubility, increase antitumor activity and decrease systemic toxicity when compared to the parent drug [68].

An interesting targeted drug containing methotrexate (chemotherapeutic agent), folic acid (targeting molecule) and fluorescein (detecting agent) was synthesized and the biological activity was assessed [13,69,70]. PAMAM, generation 5, was used as dendrimer carrier. The findings showed the success of prodrug design, since the corresponding molecule was able to bind, internalize, and induce cytotoxicity in folic acid receptor-expressing KB cells. Compounds without folic acid were less active. Similar approach was applied by Majoros and coworkers [70] fluorescein isothiocyanate (imaging agent), folic acid and paclitaxel (chemotherapeutic drug) were conjugated to PAMAM generation 5, and the molecule was characterized by different techniques, such as permeation chromatography (GPC) and nuclear magnetic resonance spectroscopy (NMR). *In vitro* and *in vivo* results were very promising in avoiding nonspecific targeting interactions, showing the importance of targeted drugs in the development of new compounds for cancer therapy.

**Figure 17:**
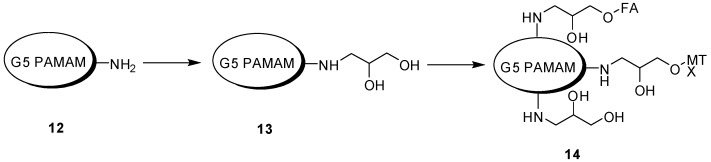
“One pot” reaction to obtain targeted prodrugs [71].

Targeted dendrimeric anticancer prodrug composed by conjugation of G5 PAMAM dendrimer, folic acid (FA) and methotrexate (MTX) (G5-FA-MTX) was synthesized by “one pot” method [71]. This synthetic method was easy, reproducible, and viable for large-scale synthesis. The amount of FA *vs.* MTX could be set to achieve the intended therapeutic effect. G5-NH_2_ (**12**) was used as starting material to obtain desired targeted dendrimer prodrug (Figure 17). Briefly, amino groups of G5-NH_2_ reacted with glycidol to obtain G5-Gly-OH (**13**). MTX and FA were coupled to the hydroxyl groups of the G5-Gly-OH (**14**) through ester bonds in a one-pot reaction, using 2-chloro-1-methylpyridinium iodide and 4-(dimethylamino)pyridine, as coupling reagents (Mukaiyama reagent). *In vitro* studies showed the same results of molecules either synthesized by conventional or “one pot” reactions. Therefore, this new approach will be helpful in obtaining targeted compounds with multiple functionalities [71,72,73]. 

The influence of folic acid in the design of targeting molecules was also investigated by Gao and colleagues [74]. G3 and G5 PAMAM dendrimer prodrugs containing ursolic acid and folic acid have been designed in order to overcome the known issues of ursolic acid, such as low water solubility and poor bioavailability. Cellular uptake assays showed that folic acid increased cellular uptake of the prodrugs by recognition of folate receptor that is overexpressed in HeLa cells. Besides that, in MTT assays and in cell cycle analysis a decreased of toxicity was observed, in comparison to the parent drug. Therefore, the results indicated ursolic acid as promising target group to enhance selectivity in cancer cells.

**Figure 18:**
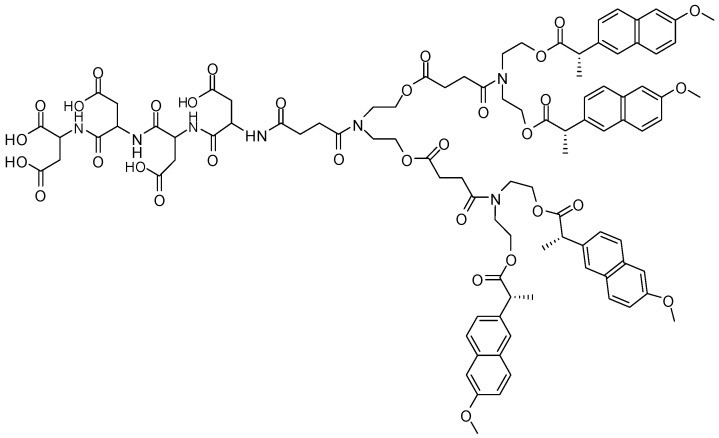
Targeted dendrimer prodrug synthesized by Pan and coworkers [75].

A series of targeted dendrimer prodrugs were synthesized to be delivered in bone [75,76] (Figure 18). The dendritic derivative was designed with naproxen and poly(aspartic acid) oligopeptide, and the structure of molecules were confirmed by NMR, mass spectral, and elemental analysis techniques. *In vitro* assays showed high affinity to hydroxyapatite, characterizing these molecules as promising for bone targeting.

Methotrexate was also used as drug standard in the design of new anticancer targeted derivatives. Goonewardena and coworkers [77] applied copper-free click chemistry to synthesize their molecules. The authors linked the biological agent to PAMAM generation 5 through either ester or amide bond. Additionally, the dendrimeric architecture included or not folic acid, which is recognized by the folate receptor. The main objective was to evaluate the therapeutic efficacy, since folic acid could provide a specific cell delivery of methotrexate. One of the results pointed out the difference between the cytotoxicity of targeted drug and the parent drug. The compounds containing ester bond were more cytotoxic that the corresponding molecules synthesized with amide.

According to Kojima and colleagues [78,79] “metastatic cancer cells degrade extracellular matrices containing collagen”. This information was useful toward designing new prodrugs embedded in collagen gels potentially active in cancer. Thus, the doxorubicin derivatives were synthesized with different surfaces, for instance, collagen peptides and polyethylene glycol. Additionally, prodrug comprising linear poly(glutamic acid) was considered. The compounds showed differences concerning efficiency and selectivity, probably, related to their chemical structure diversity. Additionally, the authors pointed out the importance of these dendrimer prodrug/collagen hybrid gel, since these compounds decreased tumor growth, as well as reduced metastatic activity *in vivo*.

**Figure 19:**
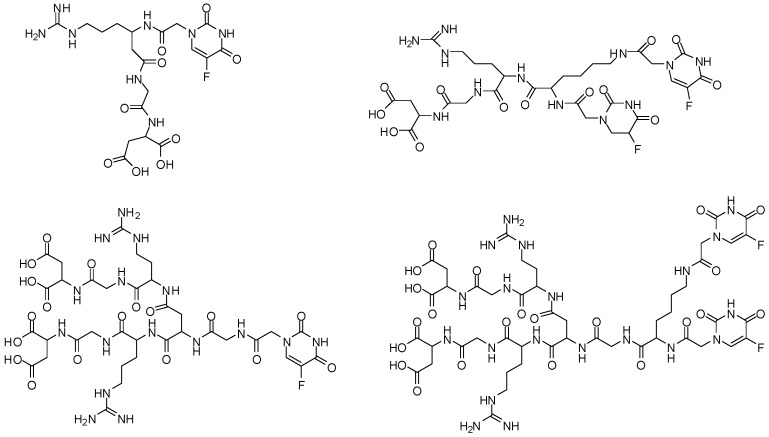
Janus peptide dendrimer selective for bone cancer therapy [80].

Janus dendrimers are composed by two dendrons with different types of functional groups located at periphery, and/or the core, and/or inner layers. Thus, those dendrimers may be helpful for improving the pharmacokinetic properties, due to the attachment of several molecules, such as bioactive compounds or targeting moieties. Considering that, Janus peptide dendrimer was built up using dimer of RGD (arginine-glycine-aspartic tripeptide—directing group for hydroxyapatite, able to conjugate in bone cancer) and dimer of 5-fluorouracil (5-FU—peripheral groups, Figure 19). Interesting results were obtained in the hydroxyapatite binding assays and drug release experiments. The 5-FU conjugated with RGD dimer exhibited an increase of targeting properties. On the other hand, the conjugate of 5-FU with two dimer demonstrated improvement of targeting, as well as of releasing properties. In conclusion, this compound may decrease the side effects in normal tissues, with extended drug release [80].

**Figure 20:**
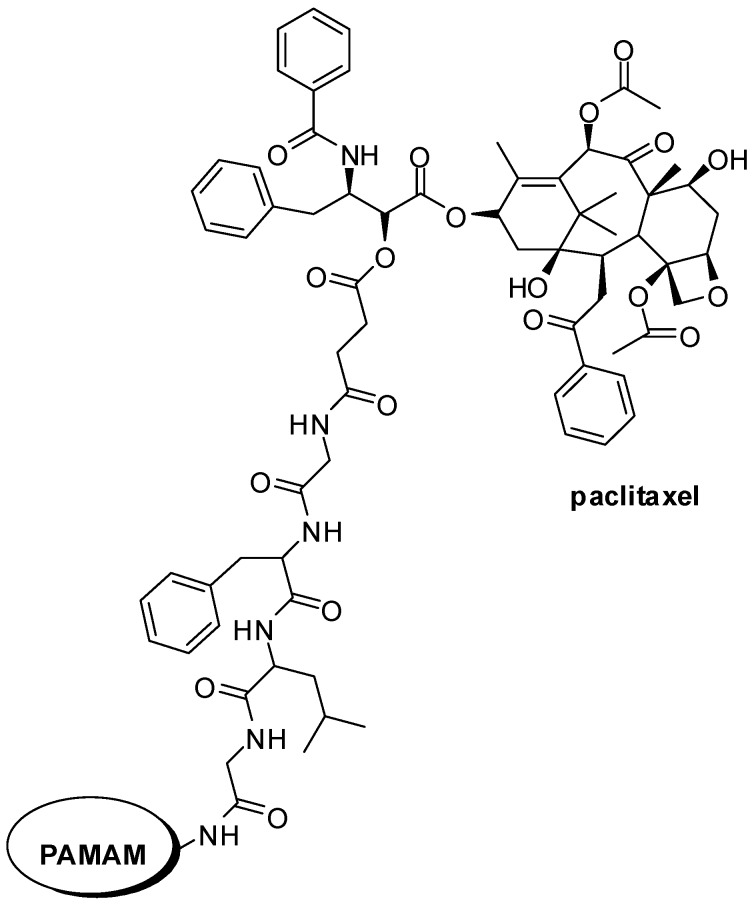
Targeted dendrimer designed for breast cancer therapy [81].

Satsangi and colleagues [81] also investigated the improvement of paclitaxel selectivity. According to the authors, the prodrug design included: (1) cathepsin B-cleavable tetrapeptide that conjugates paclitaxel to a PAMAM; and (2) the encapsulation of this dendrimer prodrug in an outer core of a RES-evading, folate receptor (FR)-targeting liposome (Figure 20). Those compounds showed better drug retention profile, increased of cytotoxicity and higher tumour size reduction, when compared with traditional targeted liposomes [81,82].

Lidicky and coworkers [83] developed doxorubicin (DOX) dendrimer and dendrimer containing DOX and rituximab (anti-CD20 monoclonal antibody—targeted group and pro-apoptotic drug). DOX and polymer were conjugated through hydrazone bonds, which are pH-sensitive hydrolytically unstable bonds, allowing controlled drug release. Conjugates were evaluated by physicochemical and biological properties (for instance, molecular weight; *in vitro* release and cytotoxicity; targeting ability; binding efficiency to CD20-cells and *in vivo* anticancer activity). *In vivo* assays with cell-based murine xenograft models of B-cell lymphoma revealed higher anti-lymphoma activity of the DOX-dendrimer, when compared to the parent drug. There was not increase of the antitumor activity in dendrimers composed by DOX and rituximab. The authors believe that there may have been changes in the binding capacity to the CD-20 receptor or loss of immunological property in conjugate containing rituximab [83].

### 2.6. Solving Problems Related to the Physicochemical Properties and Pharmaceutical Formulations

**Figure 21:**
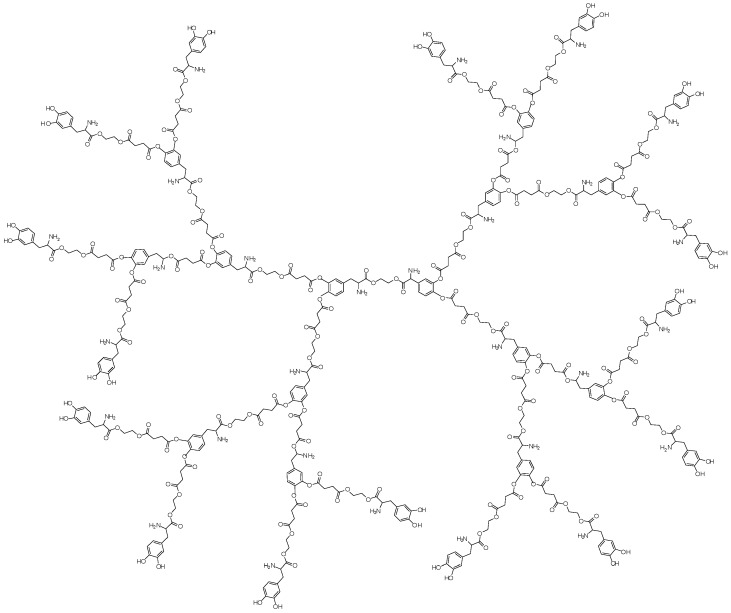
Dendrimer prodrug designed with L-dopa [15].

Dendrimer prodrugs can also be helpful for improving drug physicochemical properties, as well as to solve problems related to the pharmaceutical formulation. Thereby, dendritic macromolecules were synthesized to increase the solubility of the parent drug, L-dopa (levodopa, 3,4-dihydroxy-l-phenylalanine) [13,15]. The third generation showed 30 molecules of L-dopa, which formed the core, branches and dendrimer periphery (Figure 21). Improvement of 20-fold in aqueous solubility and molecule photostability were identified.

Paclitaxel is known as a poorly soluble anticancer agent, therefore, prodrug design could be useful to solve this problem. Hence, the compound was covalently attached in PAMAM generation 4 hydroxyl-terminated and *bis*(PEG) polymer to achieve better solubility and cytotoxicity [13,84]. The corresponding molecules were characterized using different techniques and analyzed by molecular modeling, enzymatic stability assays and *in vitro* experiments, using A2780 human ovarian carcinoma cells. The results were very promising, showing, for instance, cytotoxicity improvement of dendrimer prodrug, comparatively to the parent drug.

Zhou and coworkers [85] published an interesting paper related to the synthesis and characterization of linear-dendritic copolymers 1,2-butylene oxide and ethylene oxide with different generations of PAMAM dendrimers via carbamate bonds. One of the results showed that amphiphilic conjugates were able to self-associate in aqueous buffer solution, originating spherical “flowerlike” micelles. Moreover, the researchers pointed out the importance of these series of molecules in improving drug solubility, since the drug could be encapsulated in micelle core, as well as in the cavities between poly(ethylene oxide) or dendrimer branches.

**Figure 22:**
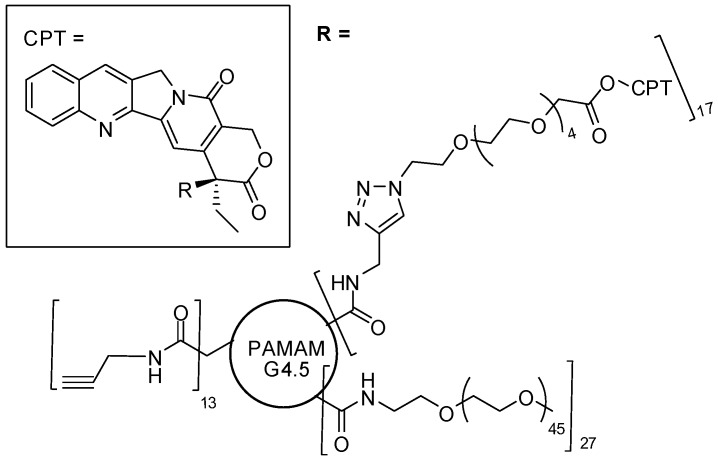
Dendrimer prodrug synthesized using click chemistry [86].

Anionic PAMAM dendrimer prodrugs for cancer therapy containing camptothecin were synthesized via click chemistry. Camptothecin was conjugated to PEGylated G4.5 PAMAM dendrimer by copper-catalyzed click reaction. This strategy improved the efficiency coupling reaction (Figure 22), as well as aimed to increase the water solubility and cytocompatibility. In human glioma U1242 assays, these compounds caused G2/M phase arrest (cellular cycle) and induced the cell death, while PEGylated dendrimer itself showed no toxicity [86].

A different and interesting approach was performed by Cai and coworkers, who designed dendrimer prodrugs for cancer treatment. Cisplatin prodrugs have been synthesized using click chemistry and paclitaxel was encapsulated in dendrimer hydrophobic cavity. Those prodrugs were named as telodendrimer. This strategy allowed a synergistic effect due to drug combination. Assays showed this synergistic effect in antitumor activity, decreasing cytotoxic effects and increasing antitumor activity, when compared to the administration of parent drugs [87].

## 3. Concluding Remarks

This brief review aimed to explore the use of dendrimer prodrugs in the development of promising compounds in different therapeutic. This review covers the period since 1985, when dendrimers were introduced by Tomalia, Newkome and colleagues. Dendrimers have offered a new approach in the area of prodrug design because of their versatility and the possibility of solving many of the problems presented by drugs/bioactive compounds, in general. Although the studies have been directed mainly to the anti-cancer area, there is a high potential for their use in other therapeutic fields. As for example, we have been designing dendrimer prodrugs for obtaining compounds potentially active on neglected tropical diseases (unpublished data).

Compared with linear polymer carriers, dendrimers show low polydispersity, allowing uniformity of prodrug structure. Besides that, the synthesis is controlled, providing compounds with regular molecular weight, predictable size, shape and three-dimensional architecture.

The excellent papers available in the literature demonstrate that dendrimer prodrugs are useful for improving the pharmacokinetic and pharmaceutical properties of prototype drugs. Thus, topics such as drug delivery, improvement of pharmacokinetic features, drug release control, decreased toxicity, as well as increase of drug selectivity were explored by means of meaningful examples in an attempt to show the great importance of these nanocarriers in drug design. Synthesis and biological evaluations have also been discussed, as well as the use of molecular modelling as a tool to assist the understanding of the molecular systems behaviour.

## References

[1] Chung M.C., Silva A.T.A., Castro L.F., Guido R.V.C., Nassute J.C., Ferreira E.I. (2005). Latenciação e formas avançadas no transporte de fármacos. Rev. Bras. Cienc. Farm..

[2] Silva A.T.A., Castro L.F., Guido R.V.C., Chung M.C., Ferreira E.I. (2005). Advances in prodrug design. Mini Rev. Med. Chem..

[3] Gardikis K., Micha-Screttas M., Steele B.R., Demetzos C. (2006). An overview of dendrimers and their biomedical applications. Pharmakeftiki.

[4] Huttunen K.M., Raunio H., Rautio J. (2011). Prodrugs—From serendipity to rational design. Pharmacol. Rev..

[5] Zawilska J.B., Wojcieszak J., Olejniczak A.B. (2013). Prodrugs: A challenge for the drug development. Pharmacol. Rep..

[6] Tomalia D.A., Baker H., Dewald J., Hall M., Kallos G., Martin S., Roeck J., Ryder J., Smith P. (1985). A new class of polymers: Starburst dendritic macromolecules. Polym. J..

[7] Newkome G.R., Yao Z.Q., Baker G.R., Gupta V.K. (1985). Micelles. Part 1. Cascade molecules. A new approach to micelles. J. Org. Chem..

[8] Kesharwani P., Jain K., Jain N.K. (2014). Dendrimer as nanocarrier for drug delivery. Prog. Polym. Sci..

[9] Tomalia D.A. (2005). Birth of a new macromolecular architecture: Dendrimers as quantized building blocks for nanoscale synthetic polymer chemistry. Prog. Polym. Sci..

[10] Ina M. (2011). Dendrimer: A novel drug delivery system. JDDT.

[11] Twibanire J.A.K., Grindley T.B. (2014). Polyester dendrimers: Smart carriers for drug delivery. Polymers.

[12] Cheng Y., Gao Y., Rao T., Li Y., Xu T. (2007). Dendrimer-based prodrugs: Design, synthesis, screening and biological evaluation. Comb. Chem. High Troughput Screen..

[13] Svenson S. (2009). Dendrimers as versatile platform in drug delivery applications. Eur. J. Pharm. Biopharm..

[14] Ferreira E.I., Giarolla J. Pró-Fármaco Dendrimérico, Processo para sua Preparação e Composições Contendo os Mesmos. http://www.patentesonline.com.br/pr-f-rmaco-dendrim-rico-processo-para-sua-prepara-o-e-composi-es-contendo-os-mesmos-199965.html.

[15] Tang S., Martinez L.J., Sharma A., Chai M. (2006). Synthesis and characterization of water-soluble and photostable L-dopa dendrimers. Org. Lett..

[16] Jevprasesphant R., Cowley J., Day N., Penny J., Attwood D., D’Emanuele A. (2006). Development of dendrimer carriers for oral drug delivery. Pharmakeftiki.

[17] Kitchens K.M., Foraker A.B., Kolhatkar R.B., Swaan P.W., Ghandehari H. (2007). Endocytosis and interaction of poly(amidoamine) dendrimers with Caco-2 cells. Pharm. Res..

[18] Ke W., Zhao Y., Huang R., Jiang C., Pei Y. (2008). Enhanced oral bioavailability of doxorubicin in a dendrimer drug delivery system. J. Pharm. Sci..

[19] Mei L., Zhang Z., Zhao L., Huang L., Yang X.L., Tang J., Feng S.S. (2013). Pharmaceutical nanotechnology for oral delivery of anticancer drugs. Adv. Drug Deliv. Rev..

[20] Jevprasesphant R., Penny J., Atwood D., D’Emanuele A. (2004). Transport of dendrimer nanocarriers through epithelial cells via the transcellular route. J. Control. Release.

[21] Jevprasesphant R., Penny J., Jala R., Atwood D., McKeown N.B., D’Emanuele A. (2003). The influence of surface modification on the cytotoxicity of PAMAM dendrimers. Int. J. Pharm..

[22] Jevprasesphant R., Penny J., Atwood D., McKeown N.B., D’Emanuele A. (2003). Engineering of dendrimer surfaces to enhance transepithelial transport and reduce cytotoxicity. Pharm. Res..

[23] El-Sayed M., Ginski M., Rhodes H., Ghandehari H. (2002). Transepithelial transport of poly(amidoamine) dendrimers across Caco-2 cell monolayers. J. Control. Release.

[24] El-Sayed M., Rhodes C.A., Ginski M., Ghandehari H. (2003). Transport mechanism(s) of poly(amidoamine) dendrimers across Caco-2 cell monolayers. Int. J. Pharm..

[25] Bhadra D., Bhadra S., Jain N.K. (2003). A PEGylated dendritic nanoparticulate carrier of fluorouracil. Int. J. Pharm..

[26] Prieto M.J., Schilrreff P., Tesoriero M.V., Morilla M.J., Romero E.L. (2008). Brain and muscle of Wistar rats are the main targets of intravenous dendrimeric sulfadiazine. Int. J. Pharm..

[27] Lo S.T., Stern S., Clogston J.D., Zheng J., Adiseshaiah P.P., Dobrovolskaia M., Lim J., Patri A., Sun X., Simanek E.E. (2010). Biological assessment of triazine dendrimers as candidate platforms for nanomedicine: Toxicological profiles, solution behavior, biodistribution, and drug release and efficacy in a PEGylated, paclitaxel construct. Mol. Pharm..

[28] Lee K.B., Shah B., Subramaniam P., Kim C. (2014). Cyclodextrin-Modified Polyamines for Delivery of Therapeutic Molecules. U.S. Patent.

[29] Choksi A., Sarojini K.V.L., Vadnal P., Dias C., Suresh P.K., Khandare J. (2013). Comparative anti-inflamatory activity of poly(amidoamine) (PAMAM) dendrimer-dexamethasone conjugates with dexamethasone-liposomes. Int. J. Pharm..

[30] Zhang Y., Xiao C., Li M., Chen J., Ding J., He C., Zhuang X., Chen X. (2013). Co-delivery of 10-hydroxycamptothecin with doxorubicin conjugated prodrugs for enhanced anticancer efficacy. Macromol. Biosci..

[31] Li J., Han Y., Chen Q., Shi H., Rehman S., Siddiq M., Ge Z., Liu S. (2014). Dual endogenous stimuli-responsive polyplex micelles as smart two-step delivery nanocarriers for deep tumor tissue penetration and combating drug resistance of cisplatin. J. Mater. Chem. B..

[32] Li J., Ke W., Li H., Zha Z., Han Y., Ge Z. (2015). Endogenous stimuli-sensitive multistage polymeric micelleplex anticancer drug delivery system for efficient tumor penetration and cellular internalization. Adv. Healthc. Mater..

[33] D’Emanuele A., Jevprasesphant R., Penny J., Attwood D. (2004). The use of a dendrimer-propranolol prodrug to bypass efflux transporters and enhance oral bioavailability. J. Control. Release.

[34] Najlah M., Freeman S., Attwood D., D’Emanuele A. (2007). Synthesis and assessment of first-generation polyamidoamine dendrimer prodrugs to enhance the cellular permeability of P-GP substrates. Bioconjug. Chem..

[35] Saovapakhiran A., D’Emanuele A., Attwood D., Penny J. (2009). Surface modification of PAMAM dendrimers modulates the mechanism of cellular internalization. Bioconjug. Chem..

[36] Najlah M., Freeman S., Attwood D., D’Emanuele A. (2006). Synthesis, characterization and stability of dendrimer prodrugs. Int. J. Pharm..

[37] Najlah M., Freeman S., Attwood D., D’Emanuele A. (2007). *In vitro* evaluation of dendrimer prodrugs for oral drug delivery. Int. J. Pharm..

[38] Lee C., Gillies E.R., Fox M.E., Guillaudeu S.J., Fréchet J.M.J., Dy E.E., Szoka F.C. (2006). A single dose of doxorubicin-functionalized bow-tie dendrimer cures mice bearing C-26 colon carcinomas. Proc. Natl. Acad. Sci. USA.

[39] Liu M., Fréchet J.M.J. (1999). Preparation of water-soluble dendritic unimolecular micelles as potential drug delivery agents. Polym. Mater. Sci. Eng..

[40] Pan G., Lemmouchi Y., Akala E.O., Bakare O. (2005). Studies on PEGylated and drug-loaded PAMAM dendrimers. J. Bioact. Compat. Polym..

[41] Greenwald R.B., Choe Y.H., McGuire J., Conover C.D. (2003). Effective drug delivery by PEGylated drug conjugates. Adv. Drug Deliv. Rev..

[42] D’Emanuele A., Attwood D. (2005). Dendrimer-drug interactions. Adv. Drug Deliv. Rev..

[43] Amir R.J., Pessah N., Shamis M., Shabat D. (2003). Self-immolative dendrimers. Angew. Chem. Int. Ed. Engl..

[44] Shamis M., Lode H.N., Shabat D. (2004). Bioactivation of self-immolative dendritic prodrugs by catalytic antibody 38C2. J. Am. Chem. Soc..

[45] Wong A.D., DeWit M.A., Gillies E.R. (2012). Amplified release through the stimulus triggered degradation of self-immolative oligomers, dendrimers, and linear polymers. Adv. Drug Deliv. Rev..

[46] Gopin A., Ebner S., Attali B., Shabat D. (2006). Enzymatic activation of second-generation dendritic prodrugs: Conjugation of self-immolative dendrimers with poly(ethylene glycol) via click chemistry. Bioconj. Chem..

[47] Amir R.J., Danieli E., Shabat D. (2007). Receiver-amplifier, self-immolative dendritic device. Chemistry.

[48] Sagi A., Segal E., Satchi-Fainaro R., Shabat D. (2007). Remarkable drug-release enhancement with an elimination-based AB3 self-immolative dendritic amplifier. Bioorg. Med. Chem..

[49] Shamis M., Shabat D. (2007). Single-triggered AB6 self-immolative dendritic amplifiers. Chemistry.

[50] Avital-Shmilovici M., Shabat D. (2009). Enzymatic activation of hydrophobic self-immolative dendrimers: The effect of reporters with ionizable functional groups. Bioorg. Med. Chem. Lett..

[51] Gnaim S., Shabat D. (2014). Quinone-methide species, a gateway to functional molecular systems: From self-immolative dendrimers to long-wavelength fluorescent dyes. Acc. Chem. Res..

[52] Li S., Szalai M.L., Kevwitch R.M., McGrath D.V.J. (2003). Dendrimer disassembly by benzyl ether depolymerization. J. Am. Chem. Soc..

[53] Lim J., Chouai A., Lo S.T., Liu W., Sun X., Simanek E.E. (2009). Design, synthesis, characterization and biological evaluation of triazine dendrimers bearing paclitaxel using ester and ester/disulfide linkages. Bioconj. Chem..

[54] Lim J., Lo S.T., Hill S., Pavan G.M., Sun X., Simanek E. (2012). Antitumor activity and molecular dynamics simulations of paclitaxel-laden triazine dendrimers. Mol. Pharm..

[55] Bosnjakovic A., Mishra M.K., Ren W., Kurtoglu Y.E., Shi T., Fan D., Kannan R.M. (2011). Poly(amidoamine) dendrimer-erythromycin conjugates for drug delivery to macrophages involved in periprosthetic inflammation. Nanomedicine.

[56] Wei J., Shi J., Zhang J., He G., Pan J., He J., Zhou R., Guo L., Ouyang L. (2013). Design, synthesis and biological evaluation of enzymatically cleavable NSAIDs prodrugs derived from self-immolative dendritic scaffolds for the treatment of inflammatory diseases. Bioorg. Med. Chem..

[57] Vembu S., Pazhamalai S., Gopalakrishnan M. (2015). Potential antibacterial activity of triazine dendrimer: Synthesis and controllable drug release properties. Bioorg Med. Chem..

[58] Giarolla J., Rando D.G., Pasqualoto K.F.M., Zaim M.H., Ferreira E.I. (2010). Molecular modeling as a promising tool to study dendrimer prodrugs delivery. J. Mol. Struct. Theochem.

[59] Giarolla J., Pasqualoto K.F.M., Rando D.G., Zaim M.H., Ferreira E.I. (2012). Molecular modeling study on the disassembly of dendrimers designed as potential antichagasic and antileishmanial prodrugs. J. Mol. Model..

[60] Santos S.S., Giarolla J., Pasqualoto K.F.M., Ferreira E.I. (2013). Molecular modeling as a tool for studying the disassembly of potentially leishmanicide-targeted dendrimer. Mol. Simul..

[61] Santos S.S., Giarolla J., Pasqualoto K.F.M., Ferreira E.I. (2015). *In silico* study to analyse the disassembly of quercetin-targeted dendrimers potentially leishmanicide. Mol. Simul..

[62] Giarolla J., Pasqualoto K.F.M., Ferreira E.I. (2013). Design and exploratory data analysis of a second generation of dendrimer prodrugs potentially antichagasic and leishmanicide. Mol. Divers..

[63] Tam J.P., Lu Y.A., Yang J.L. (2002). Antimicrobial dendrimeric peptides. Eur. J. Biochem..

[64] Liang Y., Narayanasamy J., Rapp K.L., Schinazi R.F., Chu C.K. (2006). PAMAM dendrimers and branched polyethyleneglycol (nanoparticles) prodrugs of (−)-beta-d-(2R, 4R)-dioxolane-thymine (DOT) and their anti-HIV activity. Antivir. Chem. Chemother..

[65] Howell B.A., Fan D. (2009). Poly(amidoamine) dendrimer-supported organoplatinum antitumour agents. Proc. R. Soc. A.

[66] Erez R., Segal E., Miller K., Satchi-Fainaro R., Shabat D. (2009). Enhanced cytotoxicity of a polymer-drug conjugate with triple payload of paclitaxel. Bioorg. Med. Chem..

[67] Gurdag S., Khandare J., Stapels S., Matherly L.H., Kannan R.M. (2006). Activity of dendrimer-methotrexate conjugates on methotrexate-sensitive and-resistant cell lines. Bioconj. Chem..

[68] Wiwattanapatapee R., Carrero-Gomez B., Malik N., Duncan R. (2000). Anionic PAMAM dendrimers rapidly cross adult rat intestine *in vitro*: A potential oral delivery systems?. Pharm. Res..

[69] Thomas T.P., Majoros I.J., Kotlyar A., Latallo J.F.K., Bielinska A., Myc A., Baker J.R. (2005). Targeting and inhibition of cell growth by an engineered dendritic nanodevice. J. Med. Chem..

[70] Majoros I.J., Thomas T.P., Mehta C.B., Baker J.R. (2005). Poly(amidoamine) dendrimer-based multifunctional engineered nanodevice for cancer therapy. J. Med. Chem..

[71] Zhang Y., Thomas T.P., Desai A., Zong H., Leroueil P.R., Majoros I.J., Baker J.R. (2010). Targeted dendrimeric anticancer prodrug: A methotrexate-folic acid-poly(amidoamine) conjugate and a novel, rapid, “one pot” synthetic approach. Bioconj. Chem..

[72] Hong S., Leroueil P.R., Majoros I.J., Orr B.G., Baker J.R., Holl M.M.B. (2007). The binding avidity of a nanoparticle-based multivalent targeted drug delivery platform. Chem. Biol..

[73] Latallo J.F.K., Candido K.A., Cao Z.Y., Nigavekar S.S., Majoros I.J., Thomas T.P., Balogh L.P., Khan M.K., Baker J.R. (2005). Nanoparticle targeting of anticancer drug improves therapeutic response in animal model of human epithelial cancer. Cancer Res..

[74] Gao Y., Li Z., Xie X., Wang C., You J., Mo F., Jin B., Chen J., Shao J., Chen H., Jia L. (2015). Dendrimeric anticancer prodrugs for targeted delivery of ursolic acid to folate receptor-expressing cancer cells: Synthesis and biological evaluation. Eur. J. Pharm. Sci..

[75] Pan J., Ma L., Li B., Li Y., Guo L. (2012). Novel dendritic naproxen prodrugs withpoly(aspartic acid) oligopeptide: Synthesisand hydroxyapatite binding *in vitro*. Synth. Commun..

[76] Ouyang L., Huang W., He G., Guo L. (2009). Bone targeting prodrugs based on peptide dendrimers, synthesis and hydroxyapatite binding *in vitro*. Lett. Org. Chem..

[77] Goonewardena S.N., Kratz J.D., Zong H., Desai A.M., Tang S., Emery S., Baker J.R., Huang B. (2013). Design considerations for PAMAM dendrimer therapeutics. Bioorg. Med. Chem. Lett..

[78] Kojima C., Nishisaka E., Suehiro T., Watanabe K., Harada A., Goto T., Magata Y., Kono K. (2013). The synthesis and evaluation of polymer prodrug/collagen hybrid gels for delivery into metastatic cancer cells. Nanomedicine.

[79] Kojima C., Suehiro T., Watanabe K., Ogawa M., Fukuhara A., Nishisaka E., Harada A., Kono K., Inui T., Magata Y. (2013). Doxorubicin-conjugated dendrimer/collagen hybrid gels for metastasis-associated drug delivery systems. Acta Biomater..

[80] Jiang B., Zhao J., Li Y., He D., Pan J., Cao J., Guo L. (2013). Dual-targeting Janus dendrimer based peptides for bone cancer: Synthesis and preliminary biological evaluation. Lett. Org. Chem..

[81] Satsangi A., Roy S.S., Satsangi R.K., Tolcher A.W., Vadlamudi R.K., Goins B., Ong J.L. (2015). Synthesis of a novel, sequentially active-targeted drug delivery nanoplatform for breast cancer therapy. Biomaterials.

[82] Satsangi A., Roy S.S., Satsangi R.K., Vadlamudi R.K., Ong J.L. (2014). Design of a paclitaxel prodrug conjugate for active targeting of an enzyme upregulated in breast cancer cells. Mol. Pharm..

[83] Lidický O., Janousková O., Strohalm J., Alam M., Klener P., Etrych T. (2015). Anti-lymphoma efficacy comparison of anti-CD20 monoclonal antibody-targeted and non-targeted star-shaped polymer-prodrug conjugates. Molecules.

[84] Khandare J.J., Jayant S., Singh A., Chandna P., Wang Y., Vorsa N., Minko T. (2006). Dendrimer *versus* linear conjugate: Influence of polymeric architecture on the delivery and anticancer effect of paclitaxel. Bioconj. Chem..

[85] Zhou Z., D’Emanuele A., Lennon K., Attwood D. (2009). Synthesis and micellization of linear-dendritic copolymers and their solubilization ability for poorly water-soluble drugs. Macromolecules.

[86] Zolotarskaya O.Y., Xu L., Valerie K., Yang H. (2015). Click synthesis of a polyamidoamine dendrimer based camptothecin prodrug. RSC Adv..

[87] Cai L., Xu G., Shi C., Guo D., Wang X., Luo J. (2015). Telodendrimer nanocarrier for co-delivery of paclitaxel and cisplatin: A synergistic combination nanotherapy of ovarian cancer treatment. Biomaterials.

